# Spontaneous and Induced Animal Models for Cancer Research

**DOI:** 10.3390/diagnostics10090660

**Published:** 2020-08-31

**Authors:** Anca Onaciu, Raluca Munteanu, Vlad Cristian Munteanu, Diana Gulei, Lajos Raduly, Richard-Ionut Feder, Radu Pirlog, Atanas G. Atanasov, Schuyler S. Korban, Alexandru Irimie, Ioana Berindan-Neagoe

**Affiliations:** 1 Research Center for Advanced Medicine - Medfuture, Iuliu Hatieganu University of Medicine and Pharmacy, 23 Marinescu Street, 400337 Cluj-Napoca, Romania; ancaonaciu@gmail.com (A.O.); muresan.raluca.andrada@gmail.com (R.M.); feder_richard@yahoo.com (R.-I.F.); 2 Department of Urology, The Oncology Institute “Prof Dr. Ion Chiricuta”, 400015 Cluj-Napoca, Romania; vladcristian.munteanu@gmail.com; 3 Department of Anatomy and Embryology, Iuliu Hatieganu University of Medicine and Pharmacy, 400012 Cluj-Napoca, Romania; 4 Research Center for Functional Genomics, Biomedicine and Translational Medicine, Iuliu Hatieganu University of Medicine and Pharmacy, 23 Marinescu Street, 400337 Cluj-Napoca, Romania; raduly.lajos78@gmail.com (L.R.); pirlog.radu@yahoo.com (R.P.); 5 Department of Morphological Sciences, “Iuliu Hatieganu” University of Medicine and Pharmacy, 400012 Cluj-Napoca, Romania; 6 Ludwig Boltzmann Institute for Digital Health and Patient Safety, Medical University of Vienna, Spitalgasse 23, 1090 Vienna, Austria; atanas.atanasov@univie.ac.at; 7 Institute of Genetics and Animal Biotechnology of the Polish Academy of Sciences, Jastrzebiec, 05-552 Magdalenka, Poland; 8 Institute of Neurobiology, Bulgarian Academy of Sciences, 23 Acad. G. Bonchev str., 1113 Sofia, Bulgaria; 9 Department of Pharmacognosy, University of Vienna, 1090 Vienna, Austria; 10 Department of Natural Resources and Environmental Sciences, University of Illinois at Urbana-Champaign, Urbana, IL 61801, USA; korban@illinois.edu; 11 11th Department of Surgical Oncology and Gynaecological Oncology, Iuliu Hatieganu University of Medicine and Pharmacy, 400015 Cluj-Napoca, Romania; airimie@umfcluj.ro; 12 Department of Surgery, The Oncology Institute Prof. Dr. Ion Chiricuta, 34–36 Republicii Street, 400015 Cluj-Napoca, Romania; 13 Department of Functional Genomics and Experimental Pathology, The Oncology Institute “Prof. Dr. Ion Chiricuta”, 34-36 Republicii Street, 400015 Cluj-Napoca, Romania

**Keywords:** cancer, therapy, models of disease, genetically modified, biomedical research, preclinical studies, avatar mice

## Abstract

Considering the complexity of the current framework in oncology, the relevance of animal models in biomedical research is critical in light of the capacity to produce valuable data with clinical translation. The laboratory mouse is the most common animal model used in cancer research due to its high adaptation to different environments, genetic variability, and physiological similarities with humans. Beginning with spontaneous mutations arising in mice colonies that allow for pursuing studies of specific pathological conditions, this area of in vivo research has significantly evolved, now capable of generating humanized mice models encompassing the human immune system in biological correlation with human tumor xenografts. Moreover, the era of genetic engineering, especially of the hijacking CRISPR/Cas9 technique, offers powerful tools in designing and developing various mouse strains. Within this article, we will cover the principal mouse models used in oncology research, beginning with behavioral science of animals vs. humans, and continuing on with genetically engineered mice, microsurgical-induced cancer models, and avatar mouse models for personalized cancer therapy. Moreover, the area of spontaneous large animal models for cancer research will be briefly presented.

## 1. Introduction

The diversity of oncological malignancies is influenced by the complex genetics and molecular signaling pathways developed by tumor cells in coordination with the tumor microenvironment [1,2,3]. Cancer research involves a thorough understanding and knowledge of disease particularities to develop efficient therapeutic strategies for future clinical use. In vitro studies offer important cellular information; however, data are limited due to our inability to mirror complex pathological interactions within a living organism. On the other hand, experimental animal models allow for conduct of in vitro analysis, and together confer more compelling results. Mice models for preclinical testing of novel therapeutic strategies for an ultimate goal towards clinical implementation are of utmost importance in current research practice. These models generally serve as bridges between in vitro testing and the heterogeneous makeup of a living organism whereby numerous interconnected cellular entities within a microenvironment will sustain a pathological state. Mice animal models are widely used in cancer research due to their low cost, availability, and diversity of immunocompetent and immunodeficient strains [4]. Over 95% of in vivo cancer studies utilize mice [5]. However, translational research is limited, and is hampered by many biological aspects, such as animal behavior and species differences that may contribute to misinterpretations of results [6].

Nowadays, in vivo studies focus on induced and spontaneous models of disease in small animals, and are more restrictive in large animals. Cancer incidence and development are a result of the interplay between external or exogenous factors, such as lifestyle and living area, and internal factors, such as genetics [7,8,9,10]. The implementation of induced cancer models has gained a lot of attention due to ease and availability of various protocols and techniques. These studies focus on physical/chemical stimuli used to induce a desired disease. Physical stimuli, such as light (irradiation) and chemical stimuli (cancer cells, tumor tissue, various genetic constructs, including viruses, homologous recombination, and gene editing) can act together to develop an animal disease model [11]. Furthermore, the most common method for inducing cancer is to use microsurgical techniques from cell suspension injection to tumor tissue engraftment. The most efficient strategy is to exploit genetic engineering to develop genetically programmed cancer models. Depending on cancer particularities, some protocols involve the use of a combination of physical and chemical factors to induce cancer in laboratory animals [4,6,12].

On the other hand, spontaneous initiation of cancer within the context of a human resembling organism can offer more translatable results. This phenomenon is usually observed in large animals, and offers important information regarding tumor development and those molecular mechanisms involved in this process. Particularly, companion animals tend to have a high incidence of cancer disease, and their response to therapy is very similar to that of humans. Other resemblances between animal and human pathological characteristics are represented by the heterogeneity of the tumor mass and tumor microenvironment, resistance to therapy, and metastasis development in other organs [13]. As these models are not considered laboratory animals, this drawback limits spontaneous cancer research studies.

## 2. Animal Behavior Research in Oncology

The founder of behavioral research is considered to be Charles Darwin, who launched a new era of animal research, moving on from studies like Ivan Pavlov’s on conditional reflexes to extended studies that led to winning the Nobel Prize in Physiology and Medicine (1973) [14].

Behavioral research is an area with potential for identifying key factors that can influence the cancer control process and prevention, but with increased interest in the area of the quality of life after diagnosis [14]. Animal models that resemble the evolution of human disease have significant relevance in understanding the influence of stress casualty in the outcome of cancer. Epidemiological studies are pointing out the fact that stress and depression might serve as determinative factors for cancer onset and outcome.

The mouse, besides its resemblance in anatomic, genetic, molecular, and biochemical conditions to a human, also shares common behavior features influenced by stress factors, emotional status, and circadian rhythms. Moreover, approximately 99% of human genes have synonyms in the mice genome, thus facilitating the pursuit of systems biology studies that integrate complex factors, such as environment, genetic background, molecular changes within a cell under pathological conditions, among others. At the same time, the behavioral resemblance between human and mice is an important factor when conducting preclinical studies for a translational value towards a patient; therefore, even if the final scope of the study consists in a novel therapy in oncology, the standard modulation of the environment (e.g., diet, housing, animal care) and inclusion of models with similar emotional features (age and sex dependent) are key factors in maintaining the translational value of a study. The same standard conditions are essential for inter-laboratory extrapolation of results, wherein behavioral feedback is crucial for assessment of a therapeutic response [15]. However, the term of a “controlled environment” is rather difficult to assess due to human intervention and likely modifications of results under these circumstances [16]. The general health condition is also a direct influencing factor on behavioral performance; therefore, physical abnormalities, illness, immobility, and wounded mice must be ruled out when considering a behavioral observation. Nowadays, various behavioral tests are used to evaluate stress-associated conditions, such as open-field test, elevated plus maze to evaluate anxiety-like behavior, and a tail suspension test for depressive-like behavior. The term of a “perfect test” is not well-suited; therefore, opportunities to identify a proper test must be available and the main goal of an experiment must be taken into consideration.

For the last 30 years, studies have progressively linked the emotional status of a patient with cancer development, wherein chronic stress positively influences cancer progression; however, there are limited data available on the impact of these factors on cancer initiation. One of the main mediators of this link is the tumor immune system escape process that is encouraged by the stress associated immune suppression [17]. In this context, strategies and tests are optimized for a better understanding of the effects of chronic stress on immunity. Stress animal models can be used to determine the correlation between inflammation and healing in cancer. For example, restraint stress tests can be performed to investigate levels and effects of various inflammatory cytokines (e.g., IL-6—a cytokine mainly associated with immunomodulatory roles related to B and T lymphocyte differentiation, but also associated with non-immune roles, such as enhancement of acute liver proteins production, modulation of nociception, activation of hypothalamic–pituitary axis, pyrogenic activity [18]) and stress hormones (norepinephrine, epinephrine, and corticosterone [19]) due to induced elevation under such conditions. Moreno-Smith et al. have comprehensively presented various mechanisms related to stress conditions and cancer progression toward metastasis; these mechanisms can be recapitulated in animal models to dynamically assess their impacts [17]. Inversely, amelioration of negative physiological factors through modulators of stress (in humans—e.g., social support) functions as a buffer of cellular stress responses, and concluding with improved clinical outcomes [17].

Mouse animal models have provided constrained data on the role of stress in therapeutic responses. All these tests are good indicators in identifying immune/inflammatory aberrations and affective-like behavior during tumor growth, and even following resection if survival is desired [20]. Aggressive behavior can modulate the immune system response in terms of circulating cytokines, tumor necrosis factor-α, and the C-reactive protein [21]. Resident-intruder test is used to classify the type of aggressive behavior. Rodent animal models can develop two different types of aggressive-related behaviors: defensive and offensive [22,23]. Defensive reaction is triggered by exposure to danger, while offensive reaction is determined by availability of resources.

Particularly, metastasis follows a precise route which includes proliferation, angiogenesis, invasion, and escape of immune system surveillance to complete the cascade [24]. To point out if in this process there is a casual relationship, studies have concentrated on stress response and its effect on different key points of this cascade. For example, in clinical studies, VEGF levels in the serum is found to be at lower amounts in subjects exposed to an environment of social support [25]. Moreover, in vivo studies have demonstrated that VEGF is modulated by neuroendocrine stress hormones (epinephrine, norepinephrine, and cortisol [26,27]), and exposure to a stressful habitat promotes the activation of sympathetic pathways via β-adrenoreceptors [28]. Increasing evidence shows that the effects of stress on tumor angiogenesis should be taken into consideration; for example, an orthotropic mouse model of ovarian cancer exposed daily to cycles of immobilization leads to a more invasive pattern of disease [29].

Most tests that are performed on tumor bearing mice concentrate on the depressive-like behavior; however, an interesting issue is that subjects can develop obsessive–compulsive-like conduct [30].

Circadian deregulation is a consequence of the fluctuation of stress-associated hormones and might be a crucial step in deciphering association between stress and cancer [31]. Circadian glucocorticoid rhythms can be obstructed by stress factors, thus serving as favorable bridges for tumor progression [32]. Current findings are revealing that cancer mouse models exposed to disruption of circadian cycles because of stress-related conditions, are presenting an agreeable field for tumor progression by virtue of genetic and glucocorticoids, as well as immune routes. Melatonin, a hormone produced by the pineal gland, is a factor that can inhibit cancer cell proliferation, and acts as a channel that restores circadian rhythms [33]. Melatonin is usually secreted during the night in the absence of light, and is essential for the regulation of sleep patterns and wake cycles. Decreased levels of melatonin that can be caused by light- and temperature-related stress conditions have been connected to development of human breast cancer xenografts and rat hepatomas, immunosuppression, and sleep disturbance [34,35]. On top of this, sleep disturbance on its own can lead to an increased production of cancer-stimulatory cytokines [35]. Although there are no specific associations between the development of cancer in sleep deprived individuals due to low secretion of melatonin and installation of cancer, studies have shown that melatonin is able to impair malignant cell proliferation and also initiate apoptosis [33]. A limitation in studying these features on knock-out and transgenic mouse is that these strains have a genetic background with a melatonin-deficient feature [36].

Statistical data of behavior performance of mice can be intensely mutated by the age and sex of the mice. In females, due to hormonal modifications according to their estrous cycle, the behavior can oscillate, and so it is better not to merge data between sexes [37]. Age is also a component that can alter results as some behavioral impairments can be present only in aged mice, for example loss of peripheral vision [38]. General health conditions, as mentioned above, must be integrated with good housing conditions, such as optimal temperature, humidity, systems with controlled ventilation of cages, light cycles, bedding, and environmental enrichment [39]. In this scenario, as behavioral phenotypes can be induced by a large sum of factors, collection and interpretation of data would be difficult to maintain objectively. In addition to manual registration and scoring, there are several automated systems, such as video tracking, hind paw, or subcutaneous attached magnets with advantages for non-biased measurements [40].

## 3. Mouse Models Data Bases

Selecting a favorable strain is a very challenging element of an experimental design. In this regard, there are many accessible electronic resources that are highly recommended for use. These databases provide web-based assistance which consists of a complex compendium of spontaneous and induced tumors in genetically-modified mice [41]. The Mouse Tumor Biology (MTB) database is a detailed platform that contains information about genetically defined mice (inbred, hybrid, mutant, and genetically engineered strains), genetic factors associated with tumor susceptibility in mice, somatic genetic-mutations observed in tumors, and patient-derived xenograft (PDX) models. Other relevant online data bases are presented in Table 1.

## 4. Genetically Induced Cancer Models

For oncology research, the most widely used models consist of immunocompetent or immunocompromised mice transplanted subcutaneously or orthotopically with syngeneic and xenografted tumors [53]; their use is mainly justified by their low cost and ease of generation. However, in a clinical practice, the malignant environment within an oncological patient is far more complex.

Another type of models for cancer research used in preclinical settings is represented by transgenic mice, wherein tumor suppressor genes are downregulated or oncogenes are expressed preferentially via three main methods: DNA construct microinjection, retroviral infection, and “gene-targeted transgene” method. The pioneer work for transgenic mice began in the 1980s with the study of Jaenisch and Mintz (1974) whereby they injected viral oncogenic sequences from the simian virus (SV40) into the blastocoel of mouse embryos. The adult mice did not develop malignant masses, but viral sequences were found in different types of tissues due to integration in the cellular genome [54,55]. This initial work was followed by numerous other studies to such a degree whereby in 2007, the Federation of European Laboratory Animal Science Associations established a guideline for the nomenclature of such rodent models; specifically, a transgenic rodent is considered a model that develops either spontaneous or chemically-induced mutations, or has random or targeted DNA recombination events [56]. The term of transgenic mice is used in parallel with germline genetically engineered mouse models (GEMM) [4]. Although these models surpass the tumor transplanted mice, they are still not capable of mimicking the whole expression and mutation patterns of a “carcinogenic genome”. However, the introduction of clustered regularly interspaced short palindromic repeats (CRISPR) technology has revolutionized the idea of genome editing, its complexity, and ease of generation. With the help of CRISPR, researchers can generate mice models for cancer research that can mirror complex mutational patterns, and implicitly strengthen the synonymy of the results between mice and humans.

Recently, in line with advances in immunotherapy for oncological research, humanized mouse xenograft models have been developed to functionally introduce the interplay between human cancer cells and human immune cells. These mice are transplanted with patient-derived xenografts, and also with CD34^+^ cells that will bring forward the interactions between a tumor and stromal/immune cells.

### 4.1. Methods for Generation of Transgenic Mice

**Figure 1:**
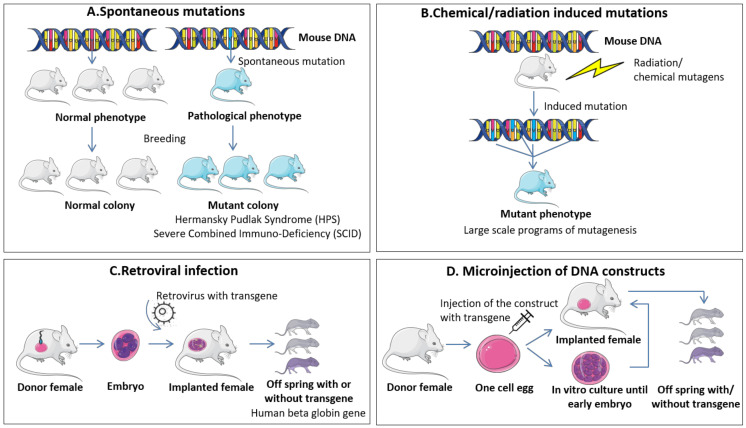
Widely used methods for generation of transgenic mice. (**A**) Spontaneous mutations. These types of modifications appear spontaneously in mice colonies after successive breeding events and are usually detected when associated with a phenotypic change. The analysis of the genetic background of spontaneously mutated mice can be associated with events encountered in human pathologies and further used as models of specific diseases. (**B**) Chemical/radiation induced mutations. These genetic modifications are based on the exposure of mice to mutagens like ethylnitrosourea (ENU) that can be used for large scale programs of mutagenesis and establishment of specific genetic alteration patterns responsible for human diseases. (**C**) Retroviral infection. This method is one of the first partially controlled protocols for generation of transgenic mice and is based on the transfection of preimplantation embryos with a retrovirus that contains the gene to be replaced/modified. The modified embryos are implanted into recipient females and analyzed for the presence or absence of the genetic modifications in concordance with the developed phenotype. (**D**) Microinjection of DNA constructs. The protocol, as the name suggests, comprises the direct injection of DNA constructs into one-cell fertilized embryos followed by transfer in recipient females and analysis of the presence or absence of the genetic modifications in concordance with the developed phenotype.

As reviewed, there are three main strategies for the generation of transgenic mice, all of which involve the introduction of DNA sequences into the genome of the mouse model. Moreover, there is a possibility of spontaneous mutations arising in mice colonies that can mimic patients’ profiles, as well as chemical- or radiation-induced mutations. The most widely used methods are schematically presented in Figure 1, and specific advantages and disadvantages are listed in Table 2.

#### 4.1.1. Spontaneous Mutations and Chemical/Radiation Induced Mutations

Among the first studies in mice genetics involved analysis of genetic mutations that spontaneously arise in breeding colonies. Some of these DNA modifications were associated with specific mutational events found in human pathologies and selectively bred to form colonies with mutated mice. The main mice models that were generated based on spontaneous mutations consist of the Hermansky Pudlak Syndrome (HPS), based on oculocutaneous pigment dilution mutations [57], and the Severe Combined Immuno-Deficiency (SCID) that are hypogammaglobulinaemic, and have severe B and T cell dysfunction that impedes their acquired immune response [58]. This last model has been extensively used since its discovery due to its compatibility with cells or tissue of foreign origin, being an ideal model for tumor xenotransplants. However, the frequency of spontaneous mutations is very low (rate of ~5 × 10−6 per locus) [59] and quite difficult to detect if no phenotypic changes occur due to specific changes in the DNA sequence (e.g., changes in the color and texture of the coat, distinct behavior, sign of illness/poor health and even death) [60]. Moreover, extensive validations are required in order to be certain that the new mutation is the accusatory factor of the modified trait. In this sense, controlled modifications of the mouse genomic make-up, termed “forward genetic” strategies, have been proven more efficient and reliable. Early events for induction of mutations consisted in exposure to radiation or chemical mutagens, such as ethylnitrosourea (ENU) [61]. The original article states that a dose of 250 mg/kg of ENU has the potency to induce a five times higher mutational rate than a dose of 600 R of x-ray. Due to its efficiency, the agent is also used nowadays for large-scale mutagenesis studies, and together with the advancement of infrastructure and techniques it is contributing to evaluation of specific patterns of gene expression of human diseases [60].

#### 4.1.2. Retroviral Infection

Retroviral DNA insertion stands among the first attempts to generate “gene-trap” approaches for transgenic mice. In 1987, Soriano and colleagues [62] infected preimplantation embryos with a recombinant retrovirus carrying the entire human beta globin gene and the bacterial neomycin phosphotransferase gene, both under the control of their own promoters. Transgenic adult mice expressed this gene construct in hematopoietic tissues. More recently, transgenic mice expressing the green fluorescent protein (GFP), either ubiquitously or tissue-specific, were produced following infection with recombinant lentiviral vectors of single-cell embryos. The control of tissue specificity was achieved by incorporating into the lentiviral vector promoters specific for certain tissues/cells—T lymphocyte-specific and muscle specific. General expression was obtained via an ubiquitous promoter capable of functioning within heterogenous types of cells. Moreover, the transgene was also transfered to the progeny [63]. However, this technique is not extensively used due to the mechanism of de novo DNA methylation that can influence expression of the viral gene following its insertion into the mice genome [60]. Other disadvantages are limitations of the gene size proportional with the capacity of the vector, and the likelihood of random integration into the mouse genome, an event that can influence the activity of neighboring genes, and producing a phenotype not specifically associated with the modified gene [4].

#### 4.1.3. Microinjection of DNA Constructs

This method was developed in 1981 when five independent research groups demonstrated the possibility of introducing exogenous DNA by microinjection into one-cell eggs [64,65,66,67,68]. This strategy, although efficient in its principle, was a “shoot from the hip” strategy as it was predisposed to numerous errors. However, years of research contributed to a final strategy whereby a mouse could be engineered to express, in time and space, a specific target gene of interest. 

The general principle of DNA microinjection technique consists of preparing a construct carrying a transgene (DNA) and a collection of one-cell fertilized embryos, followed by direct injection of the construct into these embyros, and then transfer of viable embryos into recipient synchronized pseudopregnant females. Developed mice are then analyzed for presence of the transgene [69]. Several misfolds that may occur consist of insertion of the DNA in critical spots of the genome that can generate an auxiliary mutant phenotype not specifically related to the transgene, insertion of the DNA in genome regions that are usually subjected to gene silencing, and insertion of multiple copies in tandem that can lead to recovery of an exacerbated phenotype [4,69]. However, with the introduction of CRISPR technology and an update of the injected mixture-Cas9-sgRNA-ssDNA, the specificity of DNA microinjection for genome modification has greatly increased. The use of CRISPR/Cas for genetically engineered mouse models will be discussed separately in the following sections.

#### 4.1.4. “Gene-Targeted Transgene” Method

This last method involves targeted modifications of mouse embryonic stem (ES) cells (collected from the inner cell mass of E3.5 blastocysts) from specific genetic spots by introducing mutations from a single base pair to megabase pairs at the chromosomal level. These mutations can also be specifically targeted to determine gain or loss of function [70,71,72]. In order to follow the dynamics of such genetic modifications, the donor and recipient mice of engineered stem cells differ in coat color, and will generate offsprings with patchy coat, called chimeras. Males from the chimera litter are crossed with wild-type females (control) in order to generate heterozygous mice for the mutation, followed by intercrossing to generate homozygous mutant mice (usually 25% of the litter, unless if the specific transgene is not detrimental for survival of the embryo) [70,71,72]. Considering that ES cells from the inner cell mass of E3.5 blastocysts are capable to generate any lineage of the embryo, the induced modification will be expressed ubiquitously, a parameter not necessarily advantageous for cancer research, whereby mutations arise in specific cells. For this, gene modification/inactivation can be controlled, both spatially and temporally. Cre-mediated recombination is now widely used for conditional gene deletion in a specific tissue, as the Cre recombinase of phage P1 can mediate excessive gene recombination in mammalian cells between loxP sequences (34-bp). Therefore, a DNA sequence can be specifically eliminated if encountered between two loxP via Cre-mediated recombination. Finally, for developing a tissue specific transgenic model, two general components are necessary: one mouse with a modified allele of the gene to be silenced and another mouse expressing a Cre recombinase under the control of a tissue-specific promoter. This method is described in detail by Le and Sauer [73] and by Porret and colleagues [74].

### 4.2. Models of Transgenic Mice in Concordance with the Type of Gene Modification

In general, transgenic mice can be divided into models of loss of function or gain of function.

The loss of function model is also termed knockout mice, and are highly important to investigate the activity of a specific gene in cancer development and their potential in targeted therapies. One such example is the model of E-cadherin expression in cancer progression (cause or consequence of cancer installation?). E-cadherin is one of the principal biomarkers of epithelial cells, and once lost, the cell weakens its contact with neighboring cells, and becomes migratory/invasive. This process is key for epithelial to mesenchymal transition (EMT), and it is the principal trigger for cancer metastasis. In a transgenic mouse model of pancreatic beta-cell carcinogenesis (Rip1Tag2), Perl et al. [75] showed that E-cadherin silencing overlaps with the shift from differentiated adenoma to invasive carcinoma. Moreover, crossing of these models with the ones that functionally express E-cadherin in beta-tumor cells coincides with the arrest at the adenoma phenotype, while translation of a dominant-negative E-cadherin is correlated with invasion and metastatic dissemination [75].

The gain of function model or knock-in model is mainly engaged for investigating oncogenes in relationship to cancer development.

Table 3 presents the main types of loss and gain of function transgenic mice models, together with their principal attributes and functionality in cancer research.

### 4.3. Next Generation Mouse Models for Cancer Research

Within significant advances in knowledge and infrastructure, new reliable and more cost-efficient transgenic mice have been incorporated in current research studies. These models are represented by non-germline genetically engineered mouse models (nGEMM) and alternatives in inducing DNA modifications, such as engineered nucleases [4].

nGEMMs have specific mutations only in somatic cells, and lack modifications in germline cells through the chimeric model approach or transplantation [92,93,94]. These types of mice are more reliable in terms of cancer research and are more cost effective. Extensive information about their advantages and production methods are presented by Heyer et al. [94].

For alternative DNA modifications, there are several methods with positive results: transposon-based insertional mutagenesis, Sleeping Beauty, piggyBac, RNA interference, Engineered Nucleases, and CRISPR/Cas9 System. DNA transposons are genetic elements characterized by their ability to be transferred to a different site location within the genome with the help of a transposon-encoded DNA transposase. Modifications of these transposons have led to their use as no-viral systems for genetic engineering applications in mammalian cells. One of their advantages is the capacity to mediate transpositions of single DNA segments into one or multiple sites within the host to be modified [95]. A comparative study of three different transposons, including Sleeping Beauty, Tol2, and piggyBac in terms of efficiency of stable gene transfer and number of integrants within primary T cells from the umbilical cord blood (UCB) and peripheral blood lymphocytes (PBL) demonstrated the superiority of the piggyBac system, followed by Sleeping Beauty and Tol2. Clonal expansion was enhanced in the case of Tol2 and piggyBac compared to Sleeping Beauty, feature attributed to the possibility of modified expression of cancer related genes situated within proximity of insertion sites. The integration site of the Sleeping Beauty appeared to be randomly distributed, while for the other two systems, the location was associated with DNaseI hypersensitive sites, transcriptional start sites (TSSs), or CpG islands. It was concluded that due to this final feature, Sleeping Beauty could be the optimal choice in protocols of gene transfer [96]. Within the context of generation of cell pools of recombinant cells, a side-by-side comparison between the three transposon systems showed that Sleeping Beauty and piggyBac produced a higher number of recombinant cells than Tol2; however, all three systems were proven more efficient than conventional plasmid transfection [95]. All these systems are working at fine-tuning level; however, of all of these, CRISPR/Cas9 has attracted attention due to specificity and efficiency of the DNA modification and ease of use [97]. In basic terms, CRISPR/Cas9 consists of an active nuclease, Cas9, which can cut the double-stranded DNA (ddDNA) after the single guide RNA (sgRNA) (the other key component of the system) recognizes specifically the DNA segment that should be altered. Therefore, through a controlled design of the sgRNA, CRISPR/Cas9 can target any genomic locus/loci along with minimal off-target effects, thus everything is set properly. Once the cut in the ddDNA has been made, it can be repaired by non-homologous end joining (NHEJ) or by homology-directed repair (HDR). The first form of repair usually creates either deletions or insertions into the DNA sequence that can lead to loss-of-function mutations [98]. Through CRIPR/Cas9 technologies, researchers can generate reliable models of human disease due to likelihood of including simultaneous targeted modifications within multiple sequences. This feature allows for generating authentic animal models capable of incorporating the heterogenous genetic makeup of human disease that most of the time is initiated by cumulative genetic modifications [99]. There are several early successful transgenic models for cancer research created through the incorporation of CRISPR/Cas9 system: lung cancer knock-in model via a mice model with Cas9 dependent on Cre that in the moment of sgRNA administration targets *TP53*, *Lkb1* and *K-RAS*, causing loss of function mutations for the first two genes and driver oncogenic mutations for the third gene that in the end determines the apparition of lung adenocarcinoma [100] and pancreatic cancer model with somatic mutations generated with CRISPR/Cas9 and retrograde viral vector delivery [101]. The efficiency of CRISPR/Cas9 has rapidly contributed to scaling-up of functional experiments, whereby several groups are now using high-throughput in vivo screens to identify the functionality of specific tumor suppressor genes. For example, by injecting (via AAV) large sgRNA libraries-278 sgRNAs-targeted toward known and tumor suppressor genes that are usually mutated during carcinogenesis, the authors sketched a mutational atlas for liver cancer [102]. Moreover, reinvention of the classical knockout approach by injecting both sgRNA and Cas9 mRNA into mouse zygotes has revealed between 67% and 100% efficiency, thereby significantly surpassing the efficiency of other methods, including other targeted nucleases, such as Zinc-finger nucleases (ZFNs) and transcription activator-like effector nucleases (TALENs) [103].

## 5. Microsurgical Induced Cancer Models

Genetically induced cancer models are generally time-consuming and costly, and most of the time require highly trained personnel and advanced infrastructure. Cancer mice models are also developed through local or systemic injection of malignant cells. Depending on human cancer localization, selection of the proper tumor model is based on specific disease patterns. There are two main strategies for preclinical studies: tumor cell transplantation and spontaneous initiation of cancer or host tumor implantation [5]. Microsurgical techniques are helpful to obtain experimental cancer models especially because of their availability, as well as their rapid and visible results. Therefore, tumor tissue or cancer cell suspension inoculation can vary based on the experimental design and objectives.

**Figure 2:**
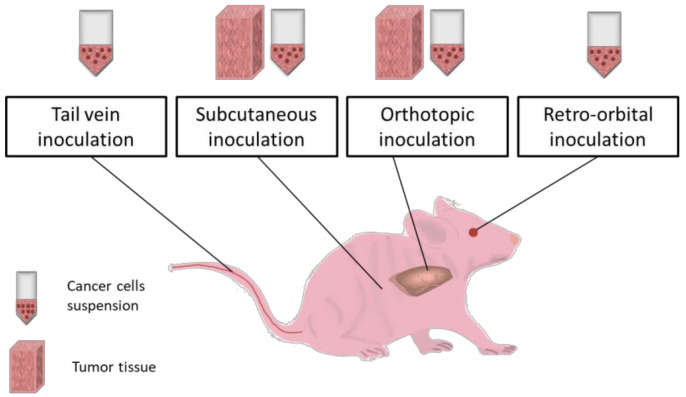
In vivo routes of cancer inoculation. Tail vein, retro-orbital, subcutaneous and orthotopic routes are suitable for cancer inoculation in laboratory mice via cancer cells suspension injection. Some of these paths (tail vein and retro-orbital) ensure minimal invasiveness and safety for the animal, providing promising results especially for cancer hematological malignancies. Moreover, tail vein mode can be engaged for treatments administration and body fluids sampling [104] while retro-orbital route is more stressful for the animal [105]. Subcutaneous implantation technique is generally used in cancer research studies because of its easiness in execution and fast results monitorization but is not prone to gain metastatic features [106]. On the other hand, orthotopic route has the capacity to mimic live organism conditions during tumor progression and can lead to metastases [107,108]. Both two routes (subcutaneous and orthotopic) are applicable for both cell suspension and tumor tissue implantation using microsurgery. Tissue samples are characterized by heterogenous cellular populations and integrity of organization level. In addition, the adequate sites for tissue implantation have large area and layered structures, and this can favor the metastatic profile allowing cells networks development and movement [109]. Subcutaneous and orthotopic routes can be explored for implantation of different electronic devices or materials which can serve as therapeutic platforms including molecular levels evaluators [110].

Figure 2 presents the most common procedures for inducing cancer in vivo in small animals like mice.

### 5.1. Subcutaneous Inoculation

The subcutaneous implantation method is used for achieving prompt tumor engraftment in order to perform tumor transplantation in a new animal, and develop a metastatic model. In addition, this procedure is suitable for studies on the cancer development process, and for some pharmacological studies. This technique is very easy to handle, economical, and provides rapid results. In brief, a cell suspension or a tumor tissue is introduced in-between the skin layer and the muscle. Tissue freshness and histological compatibility influence the inoculation success [111]. The most preferred sites for inoculation are the dorsal flanks. Studies reveal that tumor growth can be observed as of the first week after cancer cell transplantation by forming a local nodule [111]. The vascular support is an important factor in tumor growth mechanism and biological behavior, and it is supposed to influence responses to therapy [112]. In this model, therapeutic compounds present different routes of administration depending on their action pathway and effectiveness [113,114].

Moreover, tumor distant interactions can also be investigated through a subcutaneous model. A recent study shows that cidofovir therapy in human cervical carcinoma xenografts in nude mice can guide the immune system to combat cancer. Subcutaneous double xenografts are achieved through cell suspension injection. The first inoculation is made into the lower right flank, and after 3 weeks, another inoculation is made into the upper left flank of the same mouse, without physical interactions between those two sites. A Cidofovir intratumoral treatment is performed only for the tumor localized at the lower right flank, and results have indicated that the second tumor growth is affected by this treatment as well. An analysis of the immune system has demonstrated that tumor infiltrated immune cells are especially neutrophils, and their numbers decrease during therapy [115].

In subcutaneous cancer models, tumor growth monitoring is possible usually by using a digital caliper. Tumor mass measurement estimations are performed by using the following ellipsoid volume formulas: pi/6 × L × W × H and 1/2 × L × W × H [116]. Sometimes, determination of height is difficult because of tumor infiltration capacity; therefore, it is preferable to investigate the length and the width, even if this area does not necessarily correlate with tumor mass. However, tumors can be weighted after their removal. 

Despite these advantageous properties, subcutaneous injection is not capable of maintaining all the disease background encountered in humans, and therefore more complex procedures are preferred [117].

### 5.2. Orthotopic Implantation

Orthotopic engraftment represents the most favorable approach for solid tumors as it is localized at appropriate cancer primary sites, and closely mimics its histopathology and molecular patterns [118]. Therefore, disease development is similar to what happens in humans [119], and supports drug discovery platforms [120]. Compared to subcutaneous inoculation, an orthotopic procedure can induce cancer metastatic features and increases their rates through invasion and migration [121]. Unharmed tissue provides superior metastatic capacity than a cell suspension, and it is highly recommended for implantation [122]. This approach provides a spontaneous metastasis strategy for numerous cancers. For example, liver metastases are induced through spleen inoculation [123].

Tumor development in such models is monitored by weighing mice, and frequent health examinations. As tumor mass assessment cannot be performed by using digital calipers, tumor growth analysis rely on the following in vivo live imaging strategies: optical imaging (OI), computed tomography (CT), positron emission tomography (PET), single photon emission computed tomography (SPECT), magnetic resonance imaging (MRI), and ultrasonography (US) [124]. Each of these techniques is based on the use of either contrast agents or luminescent constructs, except in the US. There is a major interest in using nanoparticle optical properties to increase specificity of contrast agents [125], and for sustaining classical immunohistochemistry methods [126].

To visualize tumors, labelled cells are deemed as the best for orthotopic implantation [127,128]. There are different labeling protocols and imaging options. One of the most used labeling/imaging in standard research laboratories is a noninvasive bioluminescence imaging via assessment of the luminescent signal generated by the interaction of a luciferase enzyme (artificially introduced in the cancer cell) with its substrate (injected in the mice). Thereby, it is possible to detect, in real time, the location, spread level, and intensity of the xenograft [129].

In general, the orthotopic implantation requires direct injection of tumor cells in an organ (through imagistic guidance or taking advantage of the organ superficiality) or use of open survival surgeries for direct access of the organ. However, various non-operative strategies have been adopted based on a trans-bronchial approach. Yasufuku’s group implemented a lung cancer orthotopic model utilizing a catheter for delivering a mixture of Matrigel and cancer cell suspension into the lungs of mice. This microsurgical procedure has resulted in over 90% tumor engraftment rate, and it is deemed a minimally invasive method [130]. In some cases, orthotopic implantation is very complicated, inefficient due to tissue particularities, and mostly because of established cell lines that do not dispose of tumor specific growth behavior as in a patient [131,132]. For this reason, the subcutaneous model is more exploited. Nevertheless, orthotopic strategies are being modified to overcome these difficulties.

### 5.3. Intraperitoneal Inoculation

The intraperitoneal method can be considered a subtype of the orthotopic route as the site of implantation is specific for the development of peritoneal carcinomatosis. Peritoneal carcinomatosis is a metastatic process specific to gastrointestinal and gynecological cancers, and its occurrence jeopardizes the survival rates of patients [133]. The development of such preclinical models is very challenging, but at the same time it can be of significant value for the study of advanced forms of solid cancers. One of the difficulties is that most cells that are intraperitoneally injected are eliminated due to cellular defense processes [134].

Essentially, a cell suspension is injected into the peritoneal cavity of an animal, and tumor development is assessed through in vivo live imaging [135]. Therefore, cells used for this procedure should be luminescently labelled similar to those used for orthotopic implantation. For a successful intraperitoneal xenograft implantation, it is preferred to use Matrigel incorporated cells [136]. Extracellular matrix gel products are very efficient because they jell at 37 °C, and offer support for cell adhesion to intestines and to the peritoneum [137,138].

The inoculation of pancreatic cancer cells and tumor associated macrophages co-cultures have proved to be an efficient strategy to study pancreatic cancer metastatic features [139]. Another representative example is ovarian cancer. The intraperitoneal injection of ovarian cancer cells and fibroblast co-cultures have revealed that stromal fibroblasts are promoting ovarian cancer metastatic patterns [140].

### 5.4. Intravenous Inoculation

Intravenous induction of cancer is usually performed to develop models for metastatic cancer. The tail vein route is commonly used for such interventions; however, the success of this technique is limited by circulation turbulences, metastatic potential of cells, and by age and gender of the mice [141]. In general, cancer cell suspensions are directly injected into the tail vein of the mouse. Then, the animal is monitored through health assessment, weight monitoring, and in vivo live imaging for cancer progression. Within this context, labeled cells represent a favorable choice.

Frequently, the primary site of metastasis is localized in the lungs [142]. This occurs because lungs are rich in capillary networks and because of differences regarding systemic immunity. Masuda et al. [143] have compared systemic immunity changes between subcutaneous and intravenous administration of colon cancer cells in vivo. They have found that anti-inflammatory cytokine levels are higher in the intravenous model than in a subcutaneous model, and that these modifications will have some impact in pursuing further therapy [143].

In addition to lung metastasis animal models, some studies have relied on bone metastatic models. A widely used intra-cardiac injection has some limitations (acute death) which can be overcome via intra-caudal arterial administration [144]. This modern procedure is very efficient as cells are immediately delivered to the bone marrow site wherein they can develop metastasis. However, these cells remain on the downside of the body and form metastases, while intra-cardiac implantation achieves superior metastatic features. However, such enhanced metastatic pattern is lethal for many organs, and for this reason, the intra-caudal induction pathway is preferred. In addition, this recently discovered route can facilitate our understanding of such a complex process as bone metastasis [145].

Liver metastases have high incidence in some cancers (breast [146], colorectal [147], and prostate [148]), and it is also of interest in medical research. Breast cancer liver metastasis animal models are developed through portal vein inoculations [149]. This method is also suitable for pancreatic and colorectal adenocarcinomas, and for melanoma. Moreover, its success rate is superior to that of orthotopic implantation for such cancers.

A disadvantage of the intravenous inoculation technique is that such a models does not take into consideration the first steps of the metastatic cascade, specifically of the local primary site invasion [106]. From another standpoint, intravenous injection can represent a reliable route for inducing leukemia. In this instance, protocols recommend an irradiation step using sub-lethal X-ray doses before proceeding with leukemia cells intravenous injection [150,151].

### 5.5. Retro-Orbital Inoculation

Retro-orbital injection is extensively used as an alternative for intravascular inoculation [105] as this method is less stressful for the animal [104]. This procedure is used particularly for leukemia cell inoculation [152,153]. Moreover for this, it is recommended to sub-lethally irradiate the animals before leukemia cell inoculations [154,155]. This will ensure immune system modulation and increased engraftment efficiency [156].

Numerous studies opt for this type of implantation, not only for leukemia, but also for developing metastatic models for various cancers. A melanoma cancer lung metastatic profile was achieved via retro-orbital vein injection and followed up with in vivo live imaging [157]. This lung metastatic profile can also be obtained through retro-orbital sinus inoculation of melanoma cells [158]. The retro-orbital mode for melanoma metastases is also used for Zebrafish models [159].

Maddipati and Stanger have established a pancreatic cancer metastatic model for the lung which focuses on metastasis evolution and for progression using lineage tracing approaches [160]. They have found that the metastatic rate of cell clusters is higher than that of single-cell suspension inoculations.

In addition, the retro-orbital route is exploited for administration of different compounds required to reach the systemic vessel network [161].

Table 4 covers adequate strategies for in vivo implantation and tumor analysis, depending on the type of human cancer and its localization.

Numerous studies use a combination of the above-mentioned microsurgical implantation techniques in their protocol to establish the most efficient one for their design. Boonstra et al. have used three different cancer-inducing methods: subcutaneous injection of colorectal cancer cells, tumor orthotopic implantation in the cecum, and intraperitoneal injection of these cells to induce peritoneal carcinomatosis. The study has aimed to develop a urokinase receptor-targeted multimodal agent for non-invasive preoperative and intraoperative imaging for supporting cancer surgery [170].

Apart from inducing cancer, these routes are also used for the administration of therapeutic compounds. Novel protocols are proposing the use of implantable devices for programmed therapy in preclinical studies [171]. In this regard, intelligent nanoscale constructs, such as nanorobots, have been developed that are used for drug delivery to specific target sites in tumors based on specific molecules and their interactions with receptors [172].

## 6. Avatar Mouse Models for Personalized Cancer Therapy

### 6.1. Patient Derived Xenograft Models

Personalized medicine explores pathological conditions of individual patients to cover all information on clinical response by tailoring in vivo animal models as investigative platforms [173]. The development of avatar mouse models fosters clinical prediction and translation, and serves as important and valuable steps in medical research. The mouse model is the most widely used animal model in experimental personalized medicine. Avatar models have been developed based on PDX models. The “avatar” term has been used since the 2000s. One of the first adopters of this model is Manuel Hidalgo at Harvard Medical School (Boston, USA) who injected cells from a patient suffering from bile-duct cancer into a mouse, and studied which pharmaceuticals are best suited for treating this patient [174].

In cancer research, PDX models are basically recognized as avatar mice. They are developed through human tumor inoculations into immunocompromised mice. It is observed that these tumors can retain the human histopathological and genetic characteristics, and it can provide an important support for primary tumors expansion and for therapy prediction studies [175]. On the other hand, there are some drawbacks that limit the success of these models. For example, tumor isotype or grade, size of the tumor piece, or more specifically, the selected section from tumor tissue, together with the inoculation site in an animal are detrimental for a successful engraftment.

One proposed solution is to establish ex vivo tissue cultures, and then insert these cultures into animals. Russo et al. have studied lung cancer animal models using tumor tissue derived from five patients diagnosed with non-small-cells lung cancer: three with adenocarcinoma (AC) and two with squamous cell carcinoma (SCC). They have used 300 µm precise cut tumor tissue slices and performed 48 subrenal capsule engraftments directly or subsequently after tissue culture for 24 h. Then, mice are sacrificed at 1.5, 3, and 6 months after tumor tissue inoculation. Morphologic analysis of these new tumors has revealed that 29 of these engraftments are similar to the original cancer, while the remaining 19 engraftment present some differences resulting in an engraftment rate of 60.4%. In addition, the SCC tissue implantation have yielded an engraftment rate of 95.5%, while that for AC tumors is 30.8%. As for tissue processing, fresh or cultured tissues have no effect on engraftment rates, both approaching 100%. On the other hand, tumor volume is variable; i.e., SCC xenografts have ranged between 0.5 to 363.8 mm^3^, while AC xenografts have ranged between 0.17 to 1.36 mm^3^. In conclusion, xenograft growth in animals depends on the proliferative capacity of the primary tumor [176].

In another study, avatar models have been developed for renal cell carcinoma (RCC) derived from individual patients. Suarez et al. proposed the orthotopic implantation of primary tumor biopsies or metastasis pieces originated from 12 RCC patients into mice to test anti-angiogenic first line drugs simultaneously with second line treatments. After 5 months, the efficiency of tumor engraftment was around 75%, and tumors maintained their initial histological and metastatic properties in these avatar models. This approach supported the idea of preliminary therapy investigations in animal models as predictors for response or resistance in further clinical applications [177]. Garralda et al. reported that of 13 patient avatar models, 11 accurately mimicked a patient’s response to the disease. This finding suggested that in vivo models are feasible in providing a predictive guided therapy for clinical translation outcomes [178]. Moreover, co-clinical trials involving the patient have demonstrated the significance and the confidence of therapeutic recommendations assigned to avatar mouse models [179].

Rare cancer malignancies, such as pediatric liver cancer, have attracted the interest in developing avatar mice models for hepatoblastoma research [180,181]. Nicolle et al. have studied this condition and have developed 24 tumor derived xenografts from 20 hepatoblastomas, one hepatocellular carcinoma, two malignant rhabdoid tumors, and one transitional liver cell tumor. The metastatic tumors have grafted at a high rate of 75%, while primary tumors have achieved only a 33.3% engraftment rate. Moreover, xenografts have displayed the genomic patterns of patients with 78% of copy number variations from primary tumors present in PDXs. The responses to different treatments, including doxorubicin and cisplatin, irinotecan, and temozolomide combinations have also been investigated. It is found that drugs alone demonstrate either weak or no response, while irinotecan and temozolomide have contributed to a powerful tumor regression [182].

Considering the above-mentioned findings, PDX mice models can be validated as avatar models in cancer research platforms tailored for large-scale screening of different therapeutics [183,184]. The associations between avatar model predictions and patient observed outcomes can assess the value of in vivo models as predictors or as prognostics [185]. Furthermore, these live biological platforms ensure experimental infrastructure for understanding the correlation between histological and molecular patterns involved in tumor development, progression, invasion, and metastasis. Moreover, cancer is a genetic disease because of the specific large number of mutations discovered in such malignancies [186]. Bioinformatics analysis of tumor and normal tissue exomes can determine the most relevant mutations and corresponding targets for personalized therapies. In this regard, generating avatar mouse models through patient tumor tissue engraftment supports the study of different treatments that may have some beneficial effects against the disease. Besides providing insights in therapy response, this pre-clinical approach aids our understanding of mechanisms of resistance to therapy [179].

### 6.2. Humanized Mice Models

Real avatar models include activity of the human immune system in concordance with the presence of the disease, whereby humanized models are deemed ideal. Avatar mouse models studies have been focused on establishing a “humanized” model that embraces the human immune system or proteins together with tumor engraftment [187]. The main disadvantage is the time it takes to achieve this goal, especially in severe cases or terminal phases of disease where the therapeutic decision is crucial. Moreover, high economic expenses are a significant drawback. Even so, avatar models with defined molecular signatures can offer predictive and relevant clinical data for oncological patients in severe states wherein selection of the ideal therapeutic is crucial.

The complexity of the immune system and its implication in therapy response require extensive research, and the development of ideal avatar mouse models is very challenging [188]. Choice of the most appropriate strategy to obtain in vivo models with human immune systems strongly recommends introducing either human genes or chromosomes into a mouse genome, leading to genetic-chimera models. This technique has been utilized for Kymouse and VelocImmune mice models design. Kymouse has been obtained by using repetitive genome engineering cycles in embryonic stem cells wherein a 2.7 Mb human immunoglobulin variable-gene repertoire has been inserted leaving intact the constant regions of the mouse genome. A transgenic mouse immunization leads to the production of human antigen-specific antibodies with extended human-like complementarity reporting large epitope coverage [189]. The VelocImmune model has been developed using aVelociGene technology which involves an in situ precise genetic humanization based on bacterial artificial chromosome (BAC) targeting vectors that deliver human genes into mouse embryonic stem cells [190]. This method has allowed for replacing mouse genes with human specific genes at the same loci, and this has been used for a 6 Mb human immunoglobulin gene insertion [191]. Other studies have focused on obtaining humanized versions of innate immune cells. An MITRG mouse strain has been created with the aid of *Rag2*^−/−^ *IL2rg*^−/−^ immunodeficient mice by knocking out genes encoding a human macrophage colony stimulating factor (M-CSF), interleukin 3 (IL-3), granulocyte macrophage colony stimulating factor (GM-CSF), and thyroid peroxidase (TPO) of respective mouse loci. An MISTRG mouse strain has been obtained by including a *BAC*-transgene that encodes human signal regulatory protein alpha (SIRPα). These mice strains are used to study the macrophage infiltration in tumor xenografts resulting in human-like behavior, and can be enrolled in preclinical evaluation of therapies [192].

Although these are representing promising solutions, these models refer to weak adaptive immune responses. Immune system reconstruction with bone marrow hematopoietic stem cells could represent an advantageous alternative. In this regard, CD34^+^ hematopoietic stem cells of adult humans are integrated into a NOD/SCID/IL2 receptor γ chain null mouse grafted with allogeneic thymus tissue resulting in generation of T cells, B cells, and myeloid cells [193]. Another study proposes the generation of humanized BLT mouse involving co-transplantation of human fetal thymus, liver tissue, and CD34^+^ fetal liver cells into NOD/SCID mouse. This mouse model can generate CD4^+^ and CD8^+^ T cells in response to HIV infection leading to increased PD-1 expression [194].

To investigate human immune responses, some of the studies are focusing on immunodeficient mice because they are able to bear targeted mutations in different genes which can facilitate human primary tumors growth [195]. The targeted mutation in the IL2-receptor common gamma chain gene (*IL2rg^null^*) together with *scid*, *Rag1^null^*, or *Rag2^null^* mutations generates immunodeficiency (complete for adaptive immunity and severe for innate immunity) [196]. Therefore, NOD-Prkdc*^scid^*Il2rg*^tmiWjl^* (NSG) mouse, makes it possible to investigate the interactions between human immune response and human primary tumors. The engraftment of functional immune system is achievable especially due to IL2 gene complete mutation by hindering NK cells differentiation [197]. This represents the first successful immunodeficient mouse model especially designed for human PDX tumors and avatars for drug screening [195] and gene therapy [198]. In addition, the NSG mouse model is a promising tool for precision medicine because of the high rates of tumor engraftment and stability supporting stratification therapy [199].

Some experiments were conducted by investigating different human hematopoietic cell sources: fetal liver, umbilical cord blood, or adult blood engraftment in immunodeficient mice. Human fetal liver and umbilical cord blood represent the most relevant sources of hematopoietic stem cells while NOD-*scid*/gamma c (*−/−*) mice are the most suited strains in developing ideal humanized cell-chimera mouse models [200].

Sublethal irradiation of other immunodeficient strains prior to cell implantation (human hematopoietic stem cells from bone marrow or umbilical cord [201] or cord-blood mononuclear cells [180]) is also preferred in obtaining humanized versions of mouse models. Then, usually after a month, tumor cell lines are implanted to study and to test different immunotherapies.

**Figure 3:**
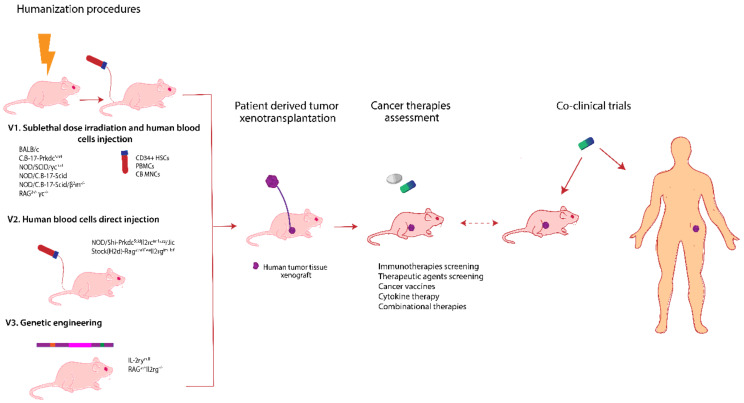
Humanized mice models development and their application in personalized cancer therapy. There are various mouse strains that can be engaged in obtaining avatar mouse models. In principle, these models are obtained following a standardized protocol (V1) involving sublethal dose irradiation and human blood cells injection: CD34^+^ human hematopoietic stem cells from bone marrow, umbilical cord (CD34^+^HSCs) [201,202], or cord blood mononuclear cells (CB MNCs) [203]. Another variant (V2) is to directly inject the peripheral blood mononuclear cells (PBMCs) [204]. A different humanization procedure involves genetic engineering (V3) and refers to knock in some human genes as *IL-3*, *M-CSF*, *GM-CSF*, thrombopoietin, and SIRPα insertion into the murine genome [192] or to knock out the genes responsible for major histocompatibility complex class I and class II molecules [205]. The next step involves human tumor tissue engraftment using microsurgery techniques. Usually the humanization process takes around 4 to 8 weeks until validation. These humanized animal models are explored to get predictive results regarding cancer therapy and can also be integrated in co-clinical trials.

Figure 3 presents in a summarized manner, the most used mouse strains for obtaining humanized models together with the methods involved in this humanization process and their applications in cancer therapy investigations.

Immune check point blockers studies on immuno-avatar animal models pose several advantages, but these are limited by xenograft versus host disease installation. Their generation involves medium complexity, high costs, and medium human relevance [206]. These models are obtained usually through peripheral blood mononuclear cells (PBMCs) inoculation in severe immunodeficient mice enabling tumor xenografts to develop in an immunological context of activated T-cells. PBMCs are implanted together with tumor cells intraperitoneally in different immunodeficient mice for achieving in vivo models of prostate cancer [207], colon cancer or gastric cancer [208]. For example, humanized non-small-cell lung cancer (NSCLC) in vivo models have been established using human PBMCs engrafted into NOD/*Scid*/IL-2Ry*^−/-^* mice. At 24h after PBMCs transfusion, CD3^+^ and CD45^+^ T cells together with antigen presenting cells population increased in the mice serum. Moreover CD3^+^ T cells are found infiltrated in lungs, liver, kidneys, spleen, and within tumor. Anti-PD-1 antibody therapy administered intraperitoneally demonstrates a reduction of tumor xenografts volume after 6 days. These promising results support the use of such avatar models in immunotherapies against lung cancer [209].

A major drawback is represented by xenograft versus host disease (xGvHD) that installs a few weeks after PBMCs inoculation, and it is believed to be associated with major histocompatibility complex (MHC) divergences between human and mouse T cells [210,211]. There are different manners to avoid this outcome. One strategy is to knock-out the genes that encode MHC class I and II molecules in mice. Another scheme involves the depletion of CD4^+^ T cells from PBMCs before inoculation and emphasizes the most promising results with highly decrease of xGvHD symptoms up to 3 months [208]. These findings denote that xGvHD is highly dependent on CD4^+^ T cells and recommend the use of immune-avatar mice models for immuno-modulatory compounds screening [206].

T cell implication is a powerful tool in the design of immune-humanized mouse models [212]. During cancer progression, tumor microenvironment modifications affects T cells biological functions by reducing their proliferative status, and overexpressing inhibitory receptors that inhibit antitumor immunity [213]. Tumor cells are prone to metabolism adaptation changes as “Warburg effect” to supply energy for cancer progression [214]. This modifications lead to immunosuppression metabolites that affect tumor infiltrating T cells (TILs) function [215]. T cells metabolic reprogramming and reactivation may represent a valuable autologous cell therapy approach with positive outcomes for patients [216,217,218].

In the application of cancer research, Jespersen et al. established a melanoma PDX model, PDXv2.0, by transplantation of tumor cells and TILs from the same patient into non-obese diabetic/severe combined immune-deficient/common gamma chain (NOG/NSG) knockout mice. Animals were treated with a recombinant human interleukin-2 according to the protocol used for patient adoptive T-cell therapy, but there were no significant effects on tumor growth. Presence of interleukin-2 influenced T-cell survival and effectiveness. Moreover, TILs were found at the tumor site, and they expressed the PD-1 surface protein. Treatment with pembrolizumab, an anti-PD-1 antibody, did not lead to the desired tumor regression effect. These findings suggested that continuous supplementation of interleukin-2 could aid TILs in tumor eradication [219]. This was a significant discovery because it offered a new perspective for on-going clinical trials and for current immuno-therapies improvement [212].

Human acute myeloid leukemia (AML) animal models can be obtained via AML blasts and cord blood-derived human progenitor cell engraftment into NSG mice. The results are visible after 6–8 weeks, and various CAR T-cell therapies can be tested [220].

Taken together, only severely immunodeficient mouse models are liable to be humanized because it is the absence of the mouse immune system that permits engraftment of a human immune system into the animal model [221]. These models are valuable for testing novel immunotherapies (immune checkpoint blockers, adoptive cell therapy, vaccines, viruses, cytokines, immunosuppressive targeting, or combinatorial therapies) in oncological malignancies [222].

## 7. Cancer Metabolism and Animal Models

A definitive feature of cancer cells is the metabolic deregulation that allows for transformed cells to proliferate at a fast rate even under restricted supply of oxygen. Therefore, malignant cells are switching to anaerobic glycolysis in exchange of mitochondrial Krebs cycle/oxidative phosphorylation system to produce energy, a process termed as the “Warburg effect”. Although this effect is mainly considered a response/feedback mechanism to lack of oxygen during tumor development, further research has revealed that this process can be responsible for tumor initiation through a series of distinct mutations in genes encoding mitochondrial metabolic enzymes: succinate dehydrogenase (SDH), isocitrate dehydrogenase (IDH), and fumarate-hydratase (FH). These three enzymes are responsible for distinct steps within the Krebs cycle [223]. In vitro models are most of the time unsuitable for such studies due to reduced numbers of mitochondria or even their absence [224], uncertain mechanisms for oxygen consumption rate [225], and lack of heterogeneity of cultured cells that cannot mimic the entire mitochondrial profile of a primary cancer [226]. For tumor biopsies, results have been obtained from an end point situation with multiple events occurring until intervention, including consequences of carcinogenesis and not necessarily causative effects [223]. For this reason, engineered mouse models harboring mutation in genes, such as *SDH*, *IDH*, and *FH* have been proposed for the systemic and dynamic analysis of tumor syndromes dependent on metabolism abnormalities (*mice with tumor cells or tissue xenografts recapitulate part of the disadvantages presented for each biological samples). These models have potentials of becoming reliable drug-testing systems; however, they fail in initiating the tumor process. This is probably due to lack of genetic “power” of these single gene changes or difference in mice genetics compared to humans. Nevertheless, they reveal important changes and connections between molecules within metabolic signaling pathways: e.g., large-scale analysis of gene expression in tissues with mutant *SDH* [227] or mutant *FH* [228]. Such approaches can identify specific mechanisms related to mitochondrial defective-induced carcinogenesis that once validated can become reliable therapeutic targets for inhibition of tumor growth and survival. Piruat et al. offer a comprehensive list and background literature of genetically engineered models of mitochondrial metabolic enzymes for oncology [223].

## 8. Spontaneous Large Animal Models for Cancer Research

**Figure 4:**
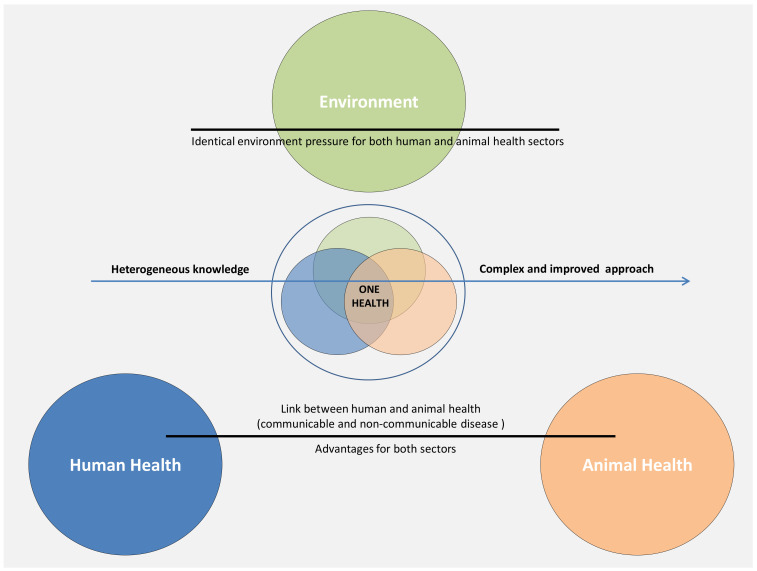
The concept of “One Health” applied to cancer studies. Although this concept is mainly applied to communicable disease affecting both humans and animals under a similar environment, the same strategy should be translated to the oncology sector due to similarities in the clinical characteristics and pathological evolution under the pressure of the same general environment.

In addition to the well-known murine models, larger animals with spontaneous cancer development can contribute to rapid advances in human and veterinary cancer therapy development [13]. These animal models are underexploited in cancer research compared to rodent models due to time, cost, and ethical reasons. Animals like canines or cats are models with high incidence of different types of cancers and present highly similar biological characteristics and response to therapy as do humans; thus, contributing to the development of reliable translational studies (Figure 4) [229]. Millions of dogs and cats are diagnosed annually with cancer worldwide and can provide good resources for cancer research [230]. Although not discussed clearly in the literature, the ethical aspects are more stringent for these animals due to close emotional contact with humans. At the same time, economic aspects are hampering adequate treatments of such animals, due to lack of public funds/programs for such type of healthcare. Therefore, inclusion of large animals with spontaneous cancers in experimental preclinical studies that in the end will actually contribute to the amelioration of symptoms will benefit both animals and humans (pathological relief for the animal and highly translational data for clinical study advancements for humans).

The critical age for cancer development in dogs is between 8–10 years corresponding with human age of 50–60 which indicates that canine cancer is age and environment dependent like in humans [13,231]. Moreover, animal malignancies resemble characteristics, such as heterogeneous mass and tumor microenvironment, resistance to therapy and metastasis development in other organs. From a histological point of view, canine tumors are very similar to human ones and have comparable response rate to chemotherapeutics [13]. The development of the dog genome sequencing project also enabled a better understanding of their genetics and implicitly allows a more precise comparison between humans and dogs in terms of genetic makeup in homeostatic and pathological states [232]. Therefore, recent studies have highlighted stringent similarities between humans and dogs with the same main tumor suppressor and oncogenes in both species responsible for cancer installation and advancement. Moreover, these correlations are more accentuated than those observed in mouse models [233,234].

Dogs can develop different type of cancers like lymphomas, skin, breast, prostate, bladder, and bone cancer, which are very similar to human cancers and can provide good research materials [234,235,236,237]. The analogy between humans and canine models arises from life style, age, food intake, and environment living conditions which are extremely comparable [229]. Therefore, the etiology and pathogenesis of domestic dogs is influenced by the same factors as humans [238]. In addition to this list of risk factors, breed characteristics are representing key factors for cancer predisposition and incidence in domestic dogs [236,239,240].

Skin cancer especially melanoma represents one of the deadliest cancers worldwide. For a better understanding of these diseases and for development of new therapeutic plans, animal models with similar characteristics to this pathology are needed. Studies demonstrate that despite of sun exposure related melanomas in humans, dog melanomas present certain characteristic resemblance from clinical presentations, histopathological features, molecular modifications, copy number alterations to protein alterations with the human skin cancer. Some melanoma-related oncogenes homologies, such *NRAS*, *BRAF*, and *V-RAS* have been reported between these two species. Canine clinical trials can serve as future solutions to new therapeutically strategies development for both humans and animals [241,242]. Besides the dog model, other animals like horses, cats, or pigs can serve as good models for skin cancer research studies. In horses, the most frequent form is represented by melanocytic tumors, and in cats, the most common form is the eye and uveal melanoma, which presents some similarities with human cutaneous alterations. These new animal models offer opportunities to identify new skin cancer related genes and therapeutic targets [243].

In recent years, several studies have proposed dogs as spontaneous animal models for mammary cancer research due to their similarities with human malignancies [244,245]. These include histopathological and molecular level alterations, as well as similar courses of the disease [246]. Specifically, both dogs and humans develop spontaneous tumors, have similar hormonal etiology, and the same approximately age of tumorigenesis onset. Other overlapping clinical characteristics are represented by tumor size development, clinical stage of the neoplasm, and lymph node metastases. Moreover, molecular similarities mainly include overexpression of steroid receptors, same proliferation markers, epidermal growth factor involvement, gene mutations, and expression patterns [247,248,249,250,251]. Significant results have also been obtained in prostate cancer [252,253], bladder cancer [237,254,255,256], bone cancer, especially osteosarcoma [257,258], and lymphomas [259,260] in terms of species (canines-humans) extrapolation for translational oncology.

## 9. Conclusions

Nowadays, cancer research is attracting high funding of research projects focused on novel early diagnostic methods and therapeutic formulations. However, what is essential for all these funding opportunities is the availability of experimental animal models useful for testing new hypotheses prior to possible clinical implementation. The resemblance of human characteristics of cancer models is directly proportional to the relevance and safety of clinical trials. These aspects have direct ethical, social, and economic impacts on our health systems, whereby a successful preclinical model can determine rapid clinical translation of results with impacts on a cancer patient’s quality of life and survival. As high as the advantages are, difficulties in generating comprehensive and relevant animal models are also as difficult. It is the capacity to mimic the human tumor evolution, a malignant microenvironment, a reaction of the organism to a testing strategy, drug metabolism, toxicity and pharmacokinetics, and other potential side effects, in concordance with a functional immune system able to accommodate the xenograft, are all characteristics that are difficult, time consuming, and expensive to mimic at once in an animal model. Additionally, when immunotherapies are now on the frontline of cancer research, advanced animal models like humanized mice are more important than ever. However, it is difficult to obtain large and homogenous cohorts of such advanced models, as it is also difficult to include relevant cohorts of large spontaneous animals to obtain significant results. Therefore, the topic of animal models for cancer research is becoming an essential area of study, with numerous investigations are yet to be made in the future. Furthermore, there should be more targeted funding for these studies, even if they are only to support models for advanced forms of preclinical investigations with no immediate results that are readily translated to a patient.

## Figures and Tables

**Table 1:** Representative animal model databases.

Data Base Name	Provided Content and Resources	Ref.
Cancer Models (caMOD)	Pathobiology with images Genetics of induced experimental models in mouse	[42]
Pathbase	Histopathology photomicrographs and macroscopic images derived from mutant or genetically manipulated mice	[43]
Cancer Genome Anatomy Project	Gene expression profiles from normal, precancerous, and cancerous tissues from mice and humans	[44]
International Mouse Strain Resource (ISMR)	Mouse strains, stocks, and mutant embryonic stem cell lines available worldwide, including inbred, mutant, and genetically engineered strains	[45]
International Mouse Phenotyping Consortium (IMPC)	The function of every protein coding gene in the mouse genome	[46]
Link Animal Models to Human Disease (LAMHDI)	The ideal animal models for different human diseases	[47]
Mouse Genome Informatics (MGI)	Integrated genetics, genomics and biological data for human health and disease studies	[48]
MUGEN mouse data base (MMdb)	Murine models of immune processes and immunological diseases	[49]
Phenotype comparisons for Disease Genes and Models (PhenoDigm)	Gene–disease associations by analyzing phenotype information	[50]
ALZFORUM	Selected rodent models of neurodegenerative disease, including Alzheimer’s, Parkinson’s, and Amyotrophic lateral sclerosis	[51]
SFARI Gene	Genes implicated in autism susceptibility	[52]

**Table 2:** Advantages and disadvantages of the widely used methods for generation of transgenic mice correspondent to Figure 1.

Method of Induction	Advantages	Disadvantages
A. Spontaneous mutations	- Discovery of novel mutations associated with specific traits/pathologies - No cost in induction of mutations	- Low mutation frequency - Hard to detect if not associated with phenotypic changes - Extensive validation to confirm the unique role of the mutation
B. Chemical/radiation induced mutations	- High mutational rate - Minimal cost for induction of mutation	- Random integrative mutations - Hard to associate specific mutations with pathologies - Extensive validation to confirm the unique role of the mutation
C. Retroviral infection	- Insertion of specific gene - Low controlled events	- De novo DNA methylation - Vector capacity in carrying large genes - Random integration in the genome
D. Microinjection of DNA constructs	- Direct insertion of specific gene - Medium controlled events - High controlled event with CRISPR/Cas9	- DNA silencing mechanisms - Insertion of multiple copies in tandem - Random integration in the genome

**Table 3:** Loss and gain of function transgenic mice models for cancer research.

	Model	Type of Gene Modification	Application	Example (Oncology)	Ref.
Loss of function	Constitutive Knockout	The gene inactivation is encountered in every cell and is also permanent	Overall changes in the phenotypical traits; identification of new genes involved in cancer	*Analyzed gene*: *DRAGO* *Function*: *p53* connected gene in response to DNA interference drugs *Model of study*: *p53^−/−^* or *p53^+/−^* mice with wild-type of deleted *Drago* (both alleles) *End point observation:* rapid tumor development and shorter survival in *p53^−/−^* or *p53^+/−^ mice with Drago deletion.*	[76,77]
	Conditional Knockout	The gene inactivation is inducible and can be time and tissue specific	Mirroring of spontaneous cancer development in a more accurate manner—at specific point during the life of the organisms and also in specific cells/tissue.	*Key components:* bacterial Cre and yeast FLP enzymes (their expression can be controlled both spatially and temporally) for recombination between specific 34-bp loxP and FRT sites that flank the gene of interest *Spatial control:* the recombinase is under the control of a tissue specific promoter *Temporal control*: tetracycline and tamoxifen-inducible systems that control the activity of Cre.	[78,79,80]
Gain of function	Constitutive Random Insertion Model	The transgene is incorporated in random spots of the genome by DNA microinjection in the pronucleus of fertilized oocytes or transfection of embryos with viral vector constructs	Activity of genes (especially oncogenes) in installation and sustenance of carcinogenesis	*Analyzed gene*: mutant *TP53* *Function:* oncogenic function and ability to sustain carcinogenesis *Model of study:* knock-in alleles with mutations that mirror the ones found in human cancers *End point observation:* mutations able to individualize the functions of apoptosis and cell cycle arrest are able to slow down the malignant development (indicating that both tasks are important for tumor suppression); each model of study (different mutated spots) exhibits a distinct phenotype, showing the complex interconnection between the dynamics of *TP53* genetics and heterogeneity of cancer.	[4,81]
	Knock-in Permissive Locus Model	Specific insertion of the gene into the genome via homologous recombination; widely used spot for insertion - *Rosa26* locus due to lack of critical genes and stable gene expression in different cellular entities	Activity of genes (especially oncogenes) in installation and sustenance of carcinogenesis	*Analyzed gene*: mutated *Npm1* (altered in AML), type A, hematopoietic compartment *Function:* *Model of study: Npm1*-TCTG/WT;Cre(+) mice generated by insertion of transgenic gene in the *Rosa26* locus with expression regulated via Cre-recombinase. *End point observation:* no development of the targeted disease (AML); perturbed megakaryocytic development and upregulation of specific miRNA profile similar to those found in humans with mutated *Npm1*: miR-10a, miR-10b, and miR-20a	[82,83,84,85]
	Conditional Knock-in Model	Adapted Constitutive Random Insertion Model, where the expression of the target gene is regulated through temporal and spatial control	Activity of genes (especially oncogenes) in installation and sustenance of carcinogenesis in a time and spatial specific manner	*Key components:* bacterial Cre and yeast FLP enzymes (their expression can be controlled both spatially and temporally) for recombination between specific 34-bp loxP and FRT sites that flank the gene of interest *Spatial control:* use of tissue specific promoters or through insertion of a STOP cassette between the promoter and the sequence of interest that is also flanked by loxP or FRT sites. Under the expression of Cre or FLP recombinase the STOP cassette is removed, and the transcription of the transgene is possible. *Temporal control:* control of Cre or FLP recombinase activity	[86,87]
	Reporter Knock-in Model	The expression of the transgene is followed by incorporation of tracking proteins—fluorescent/bioluminescent	Activity of genes (especially oncogenes) in installation and sustenance of carcinogenesis in a time and spatial specific manner and real time monitoring; tracking of tumor growth by incorporation of genes encoding tracking proteins; interaction between immune cells toward tumor inhibition	*Key components:* fluorescent proteins—e.g., GFP, RFP, bioluminescent enzymes - e.g., firefly luciferase *Exemple of model:* mice models containing firefly luciferase under the control of the human promoter E2F1, which exerts its function in proliferating cells crossed with mice models of cancer	[88,89,90,91]

*DRAGO*: drug-activated gene overexpressed; FLP: flippase; FRT: flippase recognition target; TP53: tumor protein p53; *Npm1*: nucleophosmin gene; WT: wild type; AML: acute myeloid leukemia; miRNA: microRNA; GFP: green fluorescent protein; RFP: red fluorescent protein.

**Table 4:** Selected in vivo cancer inoculation studies.

Cancer Localization	Cancer Type	Cell Line/Tissue	Animal Strain	Xenograft Method	Cancer Development Evaluation	Ref.
Skin	Melanoma	SK-mel2 and SK-mel187	Balb/c nude	Subcutaneous injection	Tumor measurements Western immunoblotting	[162]
Blood	Acute myeloid leukemia	Nalm6, Reh, Molt4 and Jurkat	NOD-*scid*-IL2Rg^−/−^ (NSI)	Irradiation followed by retro-orbital vein injection	Flow cytometry for CD45^+^ cells Bone marrow and spleen staining	[155]
	Acute lymphoblastic leukemia	Nalm-6	NOD-SCID-γc^–/–^ (NSG)	Tail vein injection	Flow cytometry Immunostaining Bioluminiscent imaging	[163]
Head and neck	Head and neck squamous cell carcinoma	FaDu, UMSCC47	Athymic nude NCI-Frederick	Mouth floor injection	Intravital imaging Immunohistochemistry	[164]
Thorax	Breast cancer	4T1-Luc MDA-MB-231	Balb/c and NSG	Subcutaneous, Intracranial and Lateral tail vein injection	In vivo imaging Immunohistochemistry	[165]
	Lewis lung carcinoma	LLC	C57BL/6 and BALB/c	Urethane intraperitoneal administration, Inferior vena cava, and subcutaneous injection	Immunohistochemistry	[166]
Abdomen	Gastric cancer	MKN-45, AGS and MKN-28	Bl6/*Rag2*/GammaC double knockout	Orthotopic injection	In vivo bioluminescence imaging Immunohistochemistry	[127]
	Pancreatic cancer	Capan-1 and SUIT-2	BALBc nu/nu	Orthotopic injection	Immunohistochemistry	[167]
Pelvis	Prostate cancer	PC-3	CB.17. SCID	Orthotopic and subcutaneous injection	Bioluminiscence Intravital microscopy Immunohistochemistry	[168]
	Epithelial ovarian cancer	Fresh tissue	BALB/c nude	Subrenal capsule implantation	Immunohistochemistry Short tandem repeat assay Western blot	[169]
